# Recent Trends of Antibiotic Resistance in *Staphylococcus aureus* Causing Clinical Mastitis in Dairy Herds in Abruzzo and Molise Regions, Italy

**DOI:** 10.3390/antibiotics12030430

**Published:** 2023-02-21

**Authors:** Franca Rossi, Ilaria Del Matto, Maria Antonietta Saletti, Luciano Ricchiuti, Patrizia Tucci, Lucio Marino

**Affiliations:** 1 Istituto Zooprofilattico Sperimentale dell’Abruzzo e del Molise, Sezione di Campobasso, 86100 Campobasso, Italy; 2 Istituto Zooprofilattico Sperimentale dell’Abruzzo e del Molise, Sezione di Lanciano, 66034 Lanciano, Italy

**Keywords:** *Staphylococcus aureus*, clinical mastitis, antibiotic resistance (AR) prevalence, AR phenotype, AR genotype, recent trend

## Abstract

This study aimed to investigate the recent trends of antibiotic resistance (AR) prevalence in *Staphylococcus aureus* isolated from the milk of animals with clinical mastitis in areas of the Abruzzo and Molise regions in Central Italy. Fifty-four *S. aureus* isolates were obtained from routine testing for clinical mastitis agents carried out in the author institution in the years 2021 and 2022 and were analyzed for phenotypic resistance to eight antibiotics recommended for testing by European norms and belonging to the antibiotic classes used for mastitis treatment in milk-producing animals. Moreover, the presence of 14 transferable genetic determinants encoding resistance to the same antibiotics was analyzed using qPCR tests developed in this study. Phenotypic resistance to non-β-lactams was infrequent, with only one 2022 isolate resistant to clindamycin. However, resistance to the β-lactam cefoxitin at concentrations just above the threshold of 4 µg/mL was observed in 59.2% of isolates in both years, making these isolates classifiable as methicillin-resistant. The AR genotypes detected were the *bla*Z gene (50% of 2021 isolates and 44.4% of 2022 isolates), *aph*A3-*bla*Z- *erm*C/T (one 2021 isolate), *aph*A3-*ant*6-*bla*Z-*erm*C/T (one 2021 isolate), *bla*Z*-erm*B (one 2022 isolate) and *mec*A-*mph* (one 2022 isolate). An inquiry into the veterinarians who provided the samples, regarding the antimicrobials prescribed for mastitis treatment and criteria of usage, indicated a possible causal relation with the AR test results. The occurrence of AR genotypes did not increase in time, most probably reflecting how mastitis was treated and prevented in farms. However, the frequently observed cefoxitin resistance needs to be explained genotypically, further monitored and limited by modifying antibiotic usage practices. The identification of a *mec*A-positive isolate in 2022 suggests further investigation if this genotype is emerging locally.

## 1. Introduction

*Staphylococcus aureus* is one of the main causative agents of mastitis in milk-producing animals and the first for clinical bovine mastitis with the ability to give causation to persistent intramammary infections [1]. This bacterial species is also a major human pathogen capable of causing food poisoning for the production of multiple heat-stable enterotoxins (SE), localized soft tissue or skin infections and systemic infections triggered by virulence factors comprising staphylococcal superantigens (SAgs) [2,3,4], cytotoxic proteins [5] and factors that favor colonization and immune evasion [6]. The emergence of antibiotic-resistant (AR) *S. aureus* strains, among which the methicillin-resistant *Staphylococcus aureus* (MRSA) are listed by the World Health Organization among the pathogens of “high priority” against which new antibiotics are urgently needed, worsens the threat to human health posed by this bacterial species, since MRSA cause infections with high mortality rates [7]. The transferable genetic element providing the MRSA phenotype is the chromosomal cassette *mec* (SCC*mec*) that most often carries the *mec*A or the *mec*C gene, and sometimes other rare homologues, encoding for additional penicillin-binding proteins (PBP2a) with reduced affinity for β-lactams plus genes for site-specific recombinases [8,9].

Use of antibiotics in the animal farming sector to treat conditions such as mastitis can cause MRSA that is transmissible to humans through raw milk and derived products [10]. Some risk factors have been identified for MRSA transmission in dairy farms, such as poor milking hygiene. However, the role of antimicrobial usage has been little investigated and only one study reported an increase in antibiotic minimum inhibitory concentration (MIC) values in the occurrence of *AR* genes *tet*K, *tet*M, and *bla*Z after enrofloxacin treatment of persistent mastitis in goats [11]. This study underlined the role of antimicrobial usage on the emergence of AR *S. aureus* strains.

Phylogenetic analysis based on multi-locus sequence typing (MLST) gives evidence that some *S. aureus* lineages are found both in human and animal hosts, in particular strains from bovine mastitis, as a consequence of transference from humans to animals and vice versa. Moreover, it was demonstrated that *S. aureus* has the capacity to switch hosts [12], therefore *S. aureus* with a resistance to antibiotics circulating among farm animals must be considered a threat to public health.

Analysis of the prevalence of antibiotic-resistant strains of *S. aureus* can indicate if risk factors that favor their increase in farms are active and allow for the adoption of measures to reduce the dissemination of the genetic determinants encoding resistance. Therefore, this study was undertaken to analyze the prevalence of AR *S. aureus* in farms by taking into account isolates from the milk of animals affected by clinical mastitis and requiring antibiotic treatment. The study was carried out in areas of the Abruzzo and Molise regions in Central Italy, and aspects of mastitis management in the sampled farms were taken into account to understand if these can be linked by a causal relation to phenotypic and genotypic AR prevalence.

## 2. Results

### 2.1. Rate of Mastitis Caused by S. aureus in 2021 and 2022

The number of farms with clinical mastitis caused by *S. aureus* were 16 among 56 analyzed in 2021 and 13 among 52 analyzed in 2022, accounting for 28.5% and 25% of mastitis outbreaks, respectively. More than one isolate was obtained from the same sample if colonies were of a different dimension and appearance, and hemolysis halo aspects were observed on the blood agar, and this led to obtaining 27 strains per year.

### 2.2. Phenotypic AR of S. aureus Isolates

**Figure 1:**
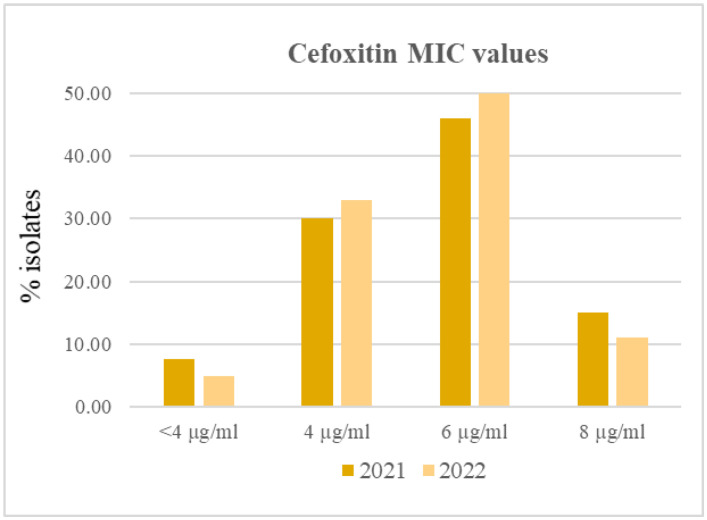
Percentages of isolates with different MIC values for cefoxitin in the years 2021 and 2022.

In this study, isolates with cefoxitin MIC 6–8 µg/mL accounted for 59.2% of isolates in both years. According to the European Committee on Antimicrobial Susceptibility Testing (EUCAST) indications, these isolates are resistant to this antibiotic, though at low levels, and must be considered methicillin-resistant [13]. However, all of them were sensitive to oxacillin. The percentages of isolates assigned to groups with different cefoxitin MIC values in the years 2021 and 2022 are shown in Figure 1.

The distributions of the *S. aureus* isolates in different cefoxitin MIC groups in the two years were highly correlated (r = 0.99), thus indicating that there was very little variation in the percentage of isolates with a given cefoxitin resistance level in the investigation period.

**Figure 2:**
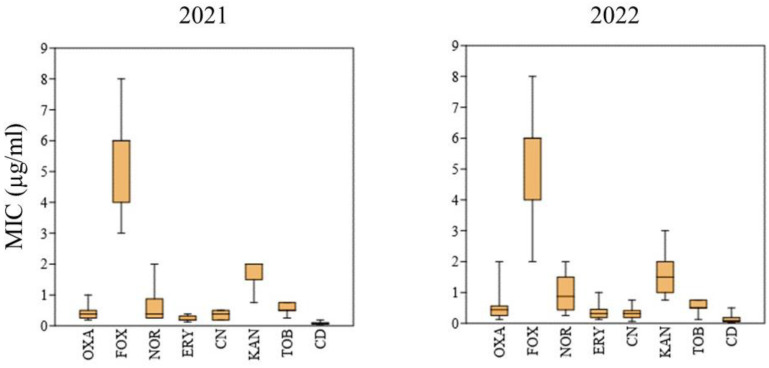
Distribution of MIC values for oxacillin (OXA), cefoxitin (FOX), norfloxacin (NOR), erythromycin (ERY), gentamicin (CN), kanamycin (KAN), tobramycin (TOB) and clindamycin (CD) in the years 2021 and 2022.

The distribution of MIC values for all antibiotics tested in the two years is shown in Figure 2.

The MIC distributions for *S. aureus* isolates between the years 2021 and 2022 were not significantly different for any of the antibiotics considered. However, it can be noted that MICs for norfloxacin showed a shift to higher values in 2022, though resistance was not detected using the disc diffusion assay. According to the MIC values, all the isolates were susceptible to oxacillin, norfloxacin, erythomycin, gentamicin, kanamycin, tobramycin and clindamycin, except for one isolate that was resistant to the latter antibiotic.

### 2.3. Occurrence of AR Genes in the S. aureus Isolates

The *AR* genes sought in this study were those encoding resistance to the antibiotics of interest and found most frequently in *S. aureus*, as deduced from the consultation of the sequence databases. In addition, the *cfr* gene was sought in this study since it codes for a 23S rRNA methyltransferase that provides resistance to different antibiotic classes, among which are lincosamides and phenicols [14]. The primer/probe systems used are reported in Table 1.

For the *erm*C/*erm*T genes and for the *mec*A/*mec*C genes, a unique primer/probe system was designed in order to merge the diagnostic tests. A *mec*A-specific test was carried out on the sole *mec*A/C positive isolate to identify the *mec* gene homolog, which was then identified as *mec*A.

The *AR* gene detected in this study and the phenotypic *AR* for each isolate are reported in Table 2.

The *bla*Z gene occurrence was frequent and found in 59.2% of 2021 isolates and in 48.1% of 2022 isolates. Differences in the occurrence of this gene between the two years were not statistically significant according to the Student’s *t* test. None of the isolates overexpressed *bla*Z to levels determining a borderline oxacillin resistant *S. aureus* (BORSA) phenotype [13].

Only a few genetic determinants beyond *bla*Z were identified in the isolates studied here. In particular, the *mec*A gene was found only in isolate six from 2022 (Table 2), which was resistant to cefoxitin (MIC 6 µg/mL) but sensitive to oxacillin, though the MIC value (2 µg/mL) was equal to the cut-off value above which *S. aureus* strains must be considered resistant [13].

Other *AR* genes occurring in the *S. aureus* isolates examined, namely *aph*3, *ant*6, *erm*B, *erm*C/T and *mph*, with the exception of *erm*B which was detected in a 2022 isolate harboring only this gene, were found mostly in association with other AR determinants. In particular, MDR genotypes *ant*6-*aph*3-*bla*Z-*erm*C/T and *aph*3-*bla*Z-*erm*C/T were each found in one 2021 isolate (Table 2).

The gene *mph* for resistance to macrolides was found in the sole *mec*A-positive strain. This strain was also resistant to clindamycin, possibly for the presence of a genetic determinant different from the genes *lnu*B and *cfr*, tested in this study.

### 2.4. Evaluation of Antibiotic Management via Veterinarian Interview

In order to understand if the results of AR screenings might be linked with a causal relation to the antibiotic usage practices adopted locally, the 18 veterinarians providing medical care to the sampled farms were interviewed via a questionnaire regarding the antibiotics used, farm hygiene and the criteria adopted for decisions surrounding antibiotic use in clinical mastitis. The results of the interviews are presented in Table 3.

It is possible to observe that all the antibiotic classes allowed for mastitis treatment were used; however, penicillins, cephalosporins and enrofloxacin prevailed.

Hygiene in the farms was considered good or acceptable in most cases, and the milking hygiene was found to be adequate in all instances. However, four veterinarians observed insufficient mastitis prevention measures in some farms. In addition, most farms were found to adopt adequate mastitis prevention measures and protocols for antibiotic usage. However, four veterinarians reported inadequate mastitis prevention measures in the farms, and these were the same veterinarians who also reported antibiotic treatment failure. Fourteen veterinarians defined farm and milking hygiene and mastitis prevention management good or adequate. Nevertheless, they reported cases of recidivating mastitis. Management of the total bacterial and somatic cell counts in bulk tank milk was referred to be good or adequate, thus showing little concern for the occurrence of subclinical mastitis.

Half of the interviewed veterinarians declared to be committed to the reduction of antibiotic usage and all of them declared to use antibiotics based on the antibiogram outcomes for the specific pathogens. Notably, all veterinarians observed cases of AR, though most of them defined those cases as rare.

## 3. Discussion

The *AR* genes sought in this study were those encoding resistance to the antibiotic classes used for mastitis therapy in dairy herds in the area of interest and found most frequently in *S. aureus*, as deduced from consultation of the sequence databases and from a recent survey on identity and the frequency of *AR* genes in 29,679 genomes of *S. aureus* isolated worldwide [16]. The qPCR technique was introduced in this study for some of the *AR* genes for which only end point PCR tests were available because they are more rapid, specific and sensitive. The design of new tests for genes for which qPCR tests were already described [17,18,19] was decided to ensure specificity for *S. aureus* after the BLASTn alignment of sequences available for the species in the public domain database. Oligonucleotides were designed to match all gene variants retrieved. In particular, for the *bla*Z and the *mec*A genes, qPCR tests were previously reported as primer/probe systems were redesigned in this study after the alignment via BLASTn of one thousand sequences for each gene. Therefore, the results can be considered comparable to those of former investigations carried out with different primer/probe systems. In addition, unique primer/probe systems were designed in this study to detect couples of genes with sequence similarity, i.e., *erm*C/*erm*T and *mec*A/*mec*C, to reduce the number of tests necessary to identify isolates with transferable AR genotypes.

The classes of β-lactams and fluoroquinolones prevailed among the antibiotics prescribed by veterinarians, and this could explain the high prevalence of strains harboring *bla*Z genes and the increase in norfloxacin MIC values in 2022. In investigations carried out in different countries, the *bla*Z gene was found at high frequencies as well, with a maximum of 95.7% [20]. The increase in norfloxacin MIC values could be indicative of an increase in resistance to quinolones that in *S. aureus* is mediated by the core gene *nor*A, encoding different variants of an efflux pump, as well as other efflux systems [14]. These are increasingly expressed under antimicrobial pressure and can lead to the emergence of resistant phenotypes [21,22].

The isolation of only one strain harboring the *mec*A gene indicated a frequency lower than that reported in other studies [23,24,25,26] but similar to that reported in Southern Italy for the bulk tank milk of small ruminants [27], thus indicating that its prevalence can vary on a local basis. The *mec*A-positive strain was not resistant to oxacillin, showing an MIC equal to the *S. aureus* epidemiological cut-off (ECOFF) for this antibiotic. The occurrence of *mec*A-positive and oxacillin-sensitive strains was reported recently [28].

The percentage of isolates resistant to cefoxitin observed in this study was among the highest reported for European countries [29]. According to the EUCAST AST guidelines, *S. aureus* strains resistant to cefoxitin have an MIC > 4 µg/mL, a value that coincides with the ECOFF of the species for cefoxitin, and in most cases they harbor a *mec*A or *mec*C gene [13]. However, in this study cefoxitin resistance was not associated with the presence of the *mec*A or the *mec*C gene, resulting in an example of a *mec*-independent β-lactam resistant phenotype. The occurrence of cefoxitin resistant isolates without the *mec* genetic determinants was described previously [30,31,32,33,34], and different genetic features were found to determine resistance only to cefoxitin with an MIC of 6 µg/mL, namely mutations in native *pbp*1, *pbp*2, *pbp*3 and *pbp*4 penicillin-binding protein genes, mutations in the *pbp*4 promoter and in gene *gdp*P for a phosphodiesterase c-di-AMP regulator [30]. Further investigations should be devoted to defining the genetic basis of cefoxitin resistance in the isolates examined in this study.

An increase in AR to clindamycin, erythromycin, gentamycin and oxacillin was not observed, though it was reported to occur globally for *S. aureus* strains, causing bovine mastitis or isolated from milk and dairy products [35,36]. In addition, a recent meta-analysis carried out in China suggested how the AR rate can be particularly high in some geographical contexts, suggesting local misuse of antibiotics [37]. Therefore, it can be hypothesized that the low frequency of phenotypic AR for some antibiotic classes observed in this study is a consequence of the low usage of those drugs, as corroborated by veterinarian’s statements. Nevertheless, isolates harboring *AR* genes encoding resistance to macrolides and aminoglycosides were identified in this study, though these were susceptible to the antibiotics for which resistance was encoded. Further experiments aimed at elucidating if those genes can be induced upon gradual exposure to antimicrobials should be carried out. Moreover, the occurrence of multiresistance-encoding mobile genetic elements should be investigated to assess the risk of MDR genotype dissemination.

According to the veterinarian statements, strains causing mastitis were isolated and tested via antibiogram only in the case of recidivating mastitis or in the case of treatment failure. This could imply that initial treatments were carried out without antibiogram execution, thus possibly leading to the selection of antibiotic resistant strains that become difficult to eradicate. Changes in this practice, together with improvements in mastitis management, could reduce the prevalence of AR *S. aureus* in farms. The answers of veterinarians to questions regarding hygiene in the farms, milking hygiene and mastitis prevention measures indicated good or acceptable levels in most cases. This might imply a low usage of antibiotics, since good farming practices reduce the occurrence of infections and need for antibiotic treatment [10]. On the other hand, the veterinarian’s statements indicated that clinical mastitis was recidivating and sometimes impossible to treat. This could be a consequence of antibiotic misusage, so the protocols of antibiotic usage adopted in farms need to be examined and, if necessary, revised to reduce the risk of AR bacteria selection.

## 4. Materials and Methods

### 4.1. Bacterial Strains and Culture Conditions

The bacterial strains used in this study were all isolates from mastitic milk samples analyzed upon the request of veterinarians by the Istituto Zooprofilattico Sperimentale dell’Abruzzo e del Molise (IZSAM), Campobasso and Lanciano branches, for identification of the infectious agent and antibiogram execution. Strains phenotypically identified as *S. aureus* in routine analysis were obtained from 56 farms and 52 farms in 2021 and 2022, respectively. These were propagated by streaking on blood agar (10/L g tryptose, 10 g/L meat extract, 5 g/L NaCl, 15 g/L agar, 100 mL of defibrinated sheep blood added aseptically after autoclaving and cooling of the base medium) incubated in aerobic conditions at 37 °C for 24–48 h. Cell biomass from a colony isolated after two subsequent streaks on blood agar was used for each phenotypic or genotypic test. For long-term storage, the isolates were maintained in Microbank (Biolife Italiana, Milan, Italy) at −80 °C.

### 4.2. Phenotypic AR Testing

The antibiotics tested phenotypically, i.e., cefoxitin (FOX), clindamycin (CD), erythromycin (ERY), gentamicin (CN), kanamycin (KAN), norfloxacin (NOR), oxacillin (OXA) and tobramycin (TOB), were those of human usage belonging to the classes of antibiotics used for mastitis treatment and recommended for testing by the EUCAST to deduce resistance to antibiotic classes [13]. The minimum inhibitory concentration (MIC) values were determined by using the Liofilchem^®^ MIC Test Strips (Liofilchem, Roseto degli Abruzzi, TE, Italy) according to the instructions. The MIC values were assigned in accordance with EUCAST guidelines on antimicrobial susceptibility testing (AST) [38]. For norfloxacin, resistance was defined by using discs with 10 µg of the antibiotic (Liofilchem) as recommended [13]. The reference to the ECOFF values [13] was used to define the position of the new isolates in the range of observed MIC values for the species *S. aureus*.

### 4.3. Quantitative PCR Primer Design

New qPCR tests for the transferable *AR* genes encoding resistance to the antibiotics tested were designed by searching and aligning sequences in the NCBI databases (https://www.ncbi.nlm.nih.gov/, accessed on 1 October 2022) and in the National Database of Antibiotic Resistant Organisms (NDARO, https://www.ncbi.nlm.nih.gov/pathogens/antimicrobial-resistance/, accessed on 2 October 2022). For each gene, a BLASTN analysis restricted to the *S. aureus* taxon was carried out in order to consider different variants to be aligned so as to design oligonucleotides targeting all of them. The primer/probe systems designed in this study are listed in Table 1 with respective target genes and amplicon dimensions. These were synthetized by Eurofins Genomics (Ebersberg, Germany).

The gene regions comprised between each pair of oligonucleotides, ranging in size between 130 and 246 bp, were synthetized upon request by GenScript Biotech (Rijswijk, The Netherlands) and delivered as pUC57 vector constructs to serve as positive controls in the qPCR runs.

### 4.4. DNA Extraction

DNA was extracted from one loopful biomass resuspended in 200 µL of Macherey Nagel T1 buffer (Carlo Erba, Cornaredo, MI, Italy) containing 100 mg of sterile 200 µm diameter glass beads in safe lock Eppendorf tubes (Eppendorf). The suspension was bead-beaten in a TissueLyser II (Qiagen Italia, Milan, Italy) at 30 hz for 2 min). Then 200 µL of Macherey Nagel B3 buffer (Carlo Erba) were added, and the extraction was continued according to the Macherey Nagel Nucleospin Tissue (Carlo Erba) protocol.

### 4.5. Quantitative PCR Conditions

The qPCR reactions were carried out in a QuantStudio 5 thermal cycler (Thermo Fisher Scientific, Rodano, MI, Italy). Identification of isolates at the species level was carried out as previously described via a *nuc*A-targeted qPCR test [39]. For AR gene detection, a unique program suitable for all the primer/probe systems designed was used. This comprised initial denaturation at 94 °C for 5 min and 40 cycles of denaturation at 94 °C for 15 s and annealing at 51 °C for 30 s. The qPCR reaction of 20 µL volume comprised 10 µL of Takara Premix Ex Taq (Probe qPCR) (Diatech, Jesi, AN, Italy), 0.2 µM primers and probe, TaqMan Exogenous Internal Positive Control Reagents (Thermo Fisher Scientific) in the recommended concentration, 2 µL of DNA sample and Nuclease Free water (Thermo Fisher Scientific) added to the final volume. Four nanograms of a synthetic, positive control construction were used in the positive control reaction.

### 4.6. Veterinarian Questionnaire

The 18 veterinarians who requested the bacteriological examinations and antibiograms for mastitis diagnosis in the years 2021 and 2022 were interviewed to identify the antibiotic classes prescribed, the criteria adopted for antibiotic usage and different aspects of mastitis management in farms by delivering a questionnaire with closed ended questions.

### 4.7. Statistical Analyses

MIC values plots, Student’s *t* test evaluation of the distinctness of the MIC data series obtained in 2021 and 2022 and correlation analyses were carried out by using PAST 4.03 free statistical software downloaded from https://past.en.lo4d.com/windows (accessed on 23 December 2022). The data series were considered distinct for *p* < 0.05.

## 5. Conclusions

This study showed that the prevalence of both genotypic and phenotypic AR is currently low for non-β-lactam antibiotics and with no increasing trend in *S. aureus* isolates from the areas of Abruzzo and Molise considered. This is probably the consequence of the infrequent usage of those antibiotics, except for enrofloxacin, reported by the veterinarians interviewed. However, strains harboring β-lactam resistance *bla*Z genes, already known to be widespread in the species *S. aureus*, occurred frequently, probably for the preferential use of β-lactams in clinical mastitis therapy. Phenotypic resistance to cefoxitin in the *mec*A/C-negative isolates was frequent, and its genetic basis needs to be identified in order to understand the molecular bases behind the emergence of this phenotype. Moreover, the occurrence of one MRSA and two genotypically MDR isolates suggest that monitoring the presence of these AR profiles in dairy herds should be continued to understand if these genotypes tend to disseminate. This study was limited to clinical mastitis cases in which the causative agent was isolated for diagnostic and antibiotic treatment purposes. In addition to the characterization of *S. aureus* strains causing clinical mastitis, investigations should be undertaken for farms with suspected subclinical mastitis to isolate the causative strains and define their AR status. This could help to elucidate if AR genotypes can be spread by strains causing subclinical mastitis. Indeed, these strains are more likely to persist for long time in farms, thus representing a relevant risk of the transfer of AR determinants.

## Figures and Tables

**Table 1:** Primers and probes designed in this study for the detection of *AR* genes, respective targets and dimensions of the amplification products.

Primer and Probe Labels and Sequences (5′-3′)	Target Gene	Amplicon Size (bp)
AadA12f: CCTGGAGAGAGCGAGA AadA12p: FAM-TTTGGAGAATGGCAGCGCAATGAC-BHQ1 AadA12r: CTATGTTCTCTTGCTTTTGT	*aad*A12	197
AadA-aph2f: GGTAGTGGTTATGATAGTG AadA-aph2p: FAM-TAGAAACTAATGTAAAAATTCCTAA-MGBEQ AadA-aph2r: TTCTGGTGTTAAAAAAGTTCC	*aad*A-*aph*2	231
Aac6f: CCTTGCGATGCTCTATG Aac6p: Cy5-CCCGACACTTGCTGACGTACA-MGBEQ Aac6r: TCCCCGCTTCCAAGAG	*aac*6 ^b^ (*aac*4)	204
Ant6f: GCGCAAATATTAATATACCTAAA Ant6P: Cy5-TGGGAATATAATAATGATG-MGBEQ Ant6r: GGGCAATAAGGTAAGATCA	*ant*6 ^b^ (*aad*E)	157
Aph3f: TGGCTGGAAGGAAAGC Aph3p: FAM-TGATGGCTGGAGCAATCTGCT-BHQ1 Aph3r: TGTCGATGGAGTGAAAGA	*aph*3-III	184
BlaZf: AAGGTTGCTGATAAAAGTGG BlaZp: FAM-GTTTATCCTAAGGGCCAATCTGAACCT-BHQ1 BlaZr: AAATTCCTTCATTACACTCTTG	*bla*Z	182
Cfrf: AAAACCTAACTGTAGATGAGA Cfrp: Cy5-GATAGCATTTCTTTTATGGGAATGGG-BHQ1 Cfrr: TAAACGAATCAAGAGCATCA	*cfr*	138
ErmAf: GGTAAACCCCTCTGAGA ErmAp: Cy5-CATCAGTACGGATATTGTC-MGBEQ ErmAr: CCCTTCTCAACGATAAGA	*erm*A	177
ErmBf: TACTCGTGTCACTTTAATTCAC ErmBp: Cy5-CAGTTTCAATTCCCTAACAAACAGAGG-BHQ1 ErmBr: CCCTAGTGTTCGGTGAA	*erm*B	205
ErmCTf: AAATGGGTTAACAAAGAATACA ErmCTp: Cy5-GAATTGACGATTTAAACAATATTAGCTTTG-BHQ1 ErmCTr: TATTGAAAAGAGACAAGAATTG	*erm*C/T ^a^	123
LnuBf: TAATTCTACCTTATCTAATCG lnuBp: FAM-GTTTAGCCAATTATCAGCAT-MGBEQ LnuBr: CGTTCATTAGAACTCTTATC	*lnu*B	113
MecAf: AGAAAAAGAAAAAAGATGGCAAA MecAp: FAM-CAACATGAAAAATGATTATGGCTCAG-BHQ1 MecAr: CTCATGCCATACATAAATGGA	*mec*A	184
mecA/Cf: ACWTCACCAGGTTCAAC mecA/Cp: Cy5-ATGGTAARGGTTGGCAAA-MGBEQ mecA/Cr: TCTGATGATTCTATTGCTTG	*mec*A*/*C ^c^	194
Mhpf: GGGACTTACATCCAGG Mphp: FAM-AAGCAAACGTCACAGGTCT-MGBEQ Mhpr: TCGTCGTCGAATACACG	*mhp*	134
MsrAF: CTTACCAATTTGAAAAAATAGCA MrsAp: Cy5-GGCAAAACCACATTACTAAATATGATTG-BHQ1 MsrAR: TTCACTCATTAAACTACCGT	*mrs*A	240

^a^ This primer/probe system targets both *erm*C and *erm*T genes; ^b^ these genes have alternative names in the sequence databases; ^c^ this primer/probe system targets both *mec*A and *mec*C genes and, coupled with the *mec*A-specific test, can allow for the detection of the *mec*C gene.

**Table 2:** List of isolates in the year 2021 or 2022 with respective AR phenotypes for the antibiotics for which resistance (R) was detected, i.e., cefoxitin (FOX) and clindamycin (CD), and the genotypic AR profiles.

2021			2022		
Farm/ Isolate *	AR Phenotype	AR Genotype	Farm/ Isolate *	AR Phenotype	AR Genotype
1			1 1	FOX R	*bla*Z
2	FOX R		1 2	FOX R	*bla*Z
3	FOX R		2 1		
4 1	FOX R	*bla*Z	2 2		
4 3	FOX R		3 1	FOX R	*bla*Z, *erm*B
5	FOX R	*aph*3, *bla*Z, *erm*CT	3 2	FOX R	
6 1	FOX R		4	FOX R	*bla*Z
6 2	FOX R		5	FOX R	
7	FOX R	*ant*6, *aph*3, *bla*Z, *erm*CT,	6	FOX R, CD R	*mec*A, *mph*
8 1	FOX R	*bla*Z	7		*bla*Z
8 2	FOX R	*bla*Z	8 1		
8 3	FOX R	*bla*Z	8 2		*bla*Z
9	FOX R		8 3		
10	FOX R		8 4		
11			9		
12	FOX R		10 1	FOX R	*bla*Z
13		*bla*Z	10 2	FOX R	*bla*Z
14 1	FOX R	*bla*Z	10 3	FOX R	*bla*Z
14 2	FOX R	*bla*Z	11 1		*bla*Z
15 1		*bla*Z	11 2		*bla*Z
15 2		*bla*Z	11 3		*bla*Z
15 3			12 1	FOX R	
16 1		*bla*Z	12 2	FOX R	*bla*Z
16 2		*bla*Z	12 3	FOX R	
16 3		*bla*Z	13 1	FOX R	
16 4		*bla*Z	13 2	FOX R	
16 5		*bla*Z	13 3	FOX R	

* The first one or two numbers indicate the farm, while the last number preceded by a space is the isolate number for cases in which more than one isolate was obtained from the same milk sample.

**Table 3:** Responses of 18 veterinarians to closed ended questions on antibiotic usage and mastitis management at farm level; all veterinarians provided medical care to the farms considered in this study. The number of veterinarians giving positive responses are reported for each question.

Question	Number of Veterinarians *
**1. Antibiotic classes prescribed**	
Aminoglycosides (gentamicin, neomicin, kanamycin)	2
Penicillins (ampicillin, amoxicillin/clavulanic acid, penicillin)	9
Cephalosporins (cefalexin, cefoperazone)	5
Lincosamides (lincomycin-spectinomycin)	2
Fluoroquinolones (enrofloxacin)	9
Macrolides (spiramycin, tylosin)	2
**2. Hygiene conditions in farms**	
Excellent	0
Good	7
Acceptable	9
Inadequate	2
**3. Milking hygiene**	
Excellent	0
Good	9
Acceptable	9
Inadequate	0
**4. Mastitis prevention measures**	
Excellent	0
Good	7
Acceptable	7
Inadequate	4
**5. Management of total bacterial and somatic cell counts in bulk tank milk** [15]	
Excellent	0
Good	13
Acceptable	5
Inadequate	0
**6. Reason for bacteriological examination and antibiogram request for mastitis cases**	
Always	0
In most cases	2
For severe infections	0
For recidivating mastitis	16
After treatment failure	4
**7. Protocol of antibiotic usage adopted**	
Always	0
In most cases	7
Frequent	7
Rare	2
None	2
**8. Evidences of AR**	
Frequent	2
Rare	16
None	0
**9. Measures adopted for AR management**	
Infectious disease expert consultation	0
Therapy against specific infectious agents	18
Reduction of antibiotic usage	9

* Some professionals gave multiple responses to questions 1, 6 and 9.

## Data Availability

Data obtained in this study can made available by authors upon request.
